# Temporal and Spatial Signatures of *Scylla paramamosain* Transcriptome Reveal Mechanistic Insights into Endogenous Ovarian Maturation under Risk of Starvation

**DOI:** 10.3390/ijms25020700

**Published:** 2024-01-05

**Authors:** Yin Fu, Fengying Zhang, Wei Wang, Jiayuan Xu, Ming Zhao, Chunyan Ma, Yongxu Cheng, Wei Chen, Zhixing Su, Xiaokang Lv, Zhiqiang Liu, Keyi Ma, Lingbo Ma

**Affiliations:** 1 Key Laboratory of East China Sea Fishery Resources Exploitation, Ministry of Agriculture and Rural Affairs, East China Sea Fisheries Research Institute, Chinese Academy of Fishery Sciences, Shanghai 200090, China; fuyin@ecsf.ac.cn (Y.F.);; 2 Centre for Research on Environmental Ecology and Fish Nutrition (CREEFFN) of the Ministry of Agriculture, Shanghai Ocean University, Shanghai 201306, China; 3 Experimental Base of East China Sea Fisheries Research Institute, Chinese Academy of Fishery Sciences, Ningbo 315604, China

**Keywords:** *Scylla paramamosain*, ovarian development, starvation, autophagy, gene expression, biochemical analysis

## Abstract

Variability in food availability leads to condition-dependent investments in reproduction. This study is aimed at understanding the metabolic response and regulatory mechanism of female *Scylla paramamosain* in response to starvation in a temporal- and tissue-specific manner. The mud crabs were starved for 7 (control), 14, 28, and 40 days for histological and biochemical analysis in the hepatopancreas, ovary, and serum, as well as for RNA sequencing on the hepatopancreas and ovary. We further highlighted candidate gene modules highly linked to physiological traits. Collectively, our observations suggested that starvation triggered endogenous ovarian maturation at the expense of hepatopancreas mass, with both metabolic adjustments to optimize energy and fatty acid supply from hepatopancreas to ovary in the early phase, followed by the activation of autophagy-related pathways in both organs over prolonged starvation. These specific adaptive responses might be considered efficient strategies to stimulate ovarian maturation of *Scylla paramamosain* under fasting stress, which improves the nutritional value of female mud crabs and other economically important crustaceans.

## 1. Introduction

Starvation is an important pressure promoting biological prosperity and species evolution [1]. Nevertheless, different species have different tolerances for starvation. Invertebrates, such as crustaceans, are smaller in size yet have survived more glacial periods compared with humans and mammals, suggesting they are capable of maintaining themselves in prolonged starving conditions [2]. For example, shrimp and crab can survive for up to two months under starvation [3,4], while aquatic subterranean crustaceans *Niphargus rhenorhodanensis* and *Niphargus virei* can starve for more than a year [5].

Crustaceans have their own strategies to tolerate food deprivation with energy-storage organs that could immediately release energy. The hepatopancreas is the major organ for the absorption and storage of nutrients such as lipids and glycogen. *Eriocheir sinensis* may resist long-term starvation owing to the important lipid storage tissues in the hepatopancreas [6]. *Macrobrachium rosenbergii* even adapts to prolonged starvation by reabsorbing organs/structures and downregulating the function of energetically costly yet unnecessary organs under food scarcity [7]. However, it remains unclear whether these reactions are due to passive consumption or the programmed regulatory strategies to fight against starvation.

A variety of reproductive attributes are also influenced by the availability of food resources. These include life history traits such as size at maturity, number of broods, fecundity, and size of eggs, as well as key reproductive processes like ovarian development and the synthesis of vitellogenin in crustaceans [8,9]. Variability in food availability is likely to lead to condition-dependent investments in reproduction [10]. Starvation constrains organisms in their allocation of energy to maintenance of body functions, somatic growth, and reproduction. A brief period of starvation has been shown to modify the lipid and protein contents in the ovary of the prawn *Penaeus monodon* [11] and to stimulate oogenesis in drosophila [12]. Whereas other studies show that during food limitation, energy allocation to maintain the basal maintenance functions (e.g., ion and acid–base regulation, ventilation, and circulation) is typically prioritized in aquatic invertebrates [13], yet growth, reproduction, or activity is reduced [14,15].

The mud crab (*Scylla paramamosain*) belongs to Crustacea, Decapoda, and Portunidae, which was originally distributed in the tropical Indo-Western Pacific region and is regarded as one of the most important economic crabs in Southeast Asia [16]. Due to its delicious meat and rich nutrition, the *Scylla* species has gained global interest, and its farming industry has rapidly expanded around the world [17]. Matured female *Scylla paramamosain* crabs whose eggs have developed to ovarian maturation stage V showed great economic value, reaching a market price twice that of a regular female crab. The first ovarian development takes 7–8 months. After laying eggs, the mud crabs do not need to mate again, and the second ovarian development takes as short as one month of cultivation.

The efforts to culture mud crabs for human consumption have prompted an interest in understanding the preferences of energy sources to be applied for feed formulation and cost reduction, where starvation studies render important insights [18]. Meanwhile, starvation has also become one of the physiological pressures that cultured crustaceans often suffer during their growth and development. Unfortunately, few studies have reported how crustaceans engage their strategies to combat starvation and the internal regulatory mechanism. Our knowledge of the effect of starvation on the reproductive organs of invertebrate species is also very limited. Moreover, whether and how starvation impacts ovarian maturation in *Scylla paramamosain* is absent. This study aims to measure the dynamics of tissue structures, material compositions, and metabolic changes in the ovary, hepatopancreas, and serum associated with both short-term and long-term starvation, along with differential transcriptomic experiments to reveal the transcriptomic differences both in different tissues and at different starvation times, in order to determine whether these transcriptomic changes cause the modification of physiological processes underlying the tolerance to starvation. Our results would provide insight into the global adaptation mechanisms in response to starvation during ovarian maturation in *Scylla paramamosain* and other economically important crustacean species.

## 2. Results

### 2.1. Maintenance of Ovarian Maturation under Imposed Starvation

**Figure 1:**
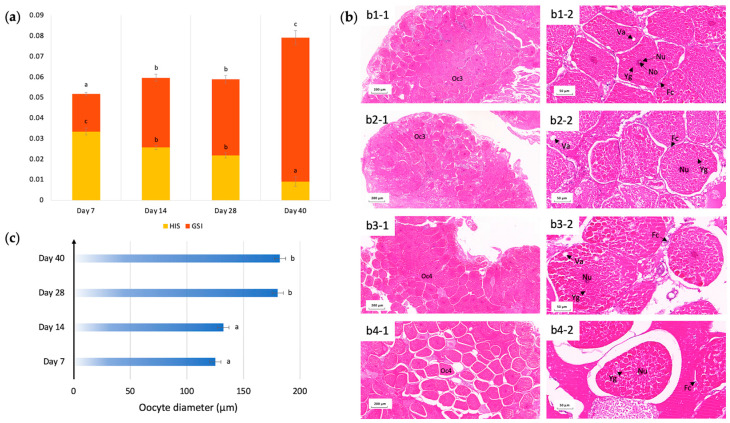
Ovarian maturation of *Scylla paramamosain* under imposed starvation. (**a**) Hepatopancreas and ovarian development under imposed starvation. Data are presented as mean ± SE (*n* = 3). GSI, gonadosomatic index; HSI, hepatosomatic index. Different letters above bars with the same color indicate significant differences (*p* < 0.05). (**b**) Histology of ovary tissues under imposed starvation at (**b1-1**) and (**b1-2**): starvation day 7; (**b2-1**) and (**b2-2**): starvation day 14; (**b3-1**) and (**b3-2**): starvation day 28; (**b4-1**) and (**b4-2**): starvation day 40. Oc3, Stage III oocytes; Oc4, Stage IV oocytes; Fc, follicular cells; Nu, nucleus; No, nucleolus; Yg, yolk granules; Va, vacuolation. (**c**) Increment of oocyte cell diameters in micrometers during prolonged starvation. Data are presented as mean ± SE (*n* = 3). Different letters indicate significant differences (*p* < 0.05).

During the experiment, the mud crabs not only survived the prolonged starvation of 40 days, but the ovarian maturation stage of *Scylla paramamosain* also successfully developed from III (days 7 and 14) to IV (days 28 and 40) against food deprivation, as characterized based on morphological and histological analysis. As the ovary tissues expanded, the gonadosomatic index (GSI) increased from 1.83% to 7.01% (Figure 1a), and the stage 3 oocytes at day 7 began to mature with an increased abundance of yolk substances, which turned predominantly into stage 4 oocytes at days 28 and 40 (Figure 1b). The increase in yolk substances was highly eosinophilic, so it was reflected by an increased intensity of redness in the cytoplasm. As the female crab reached ovarian maturation stage IV, the size of the oocytes significantly increased. The increment in size (diameter) was significant between day 14 and day 28 (*p* < 0.05), from an average of 132.3 μm (stage 3 oocytes) to 180.2 μm (stage 4 oocytes) (Figure 1c). In addition, the nucleus diameter of oocytes remained constant across stages 3–4 at around 23–25 μm (Figure 1b). Interestingly, despite the fact that the size of oocytes increased with time, the gap space between oocytes also increased dramatically with time, suggesting a loose arrangement of mature oocytes due to starvation (Figure 1b). Follicular cells were found adjacent to individual oocytes, serving as a special microenvironment for each oocyte, but were rarely seen in the large gaps surrounding oocytes at day 40, indicative of ovary shrinkage (Figure 1b).

The hepatosomatic index (HSI), on the other hand, formed an inverse relationship as the ovary matured (Figure 1a). GSI was significantly elevated with the progression of ovarian maturation under imposed starvation at the expense of the mass of the hepatopancreas. With a Pearson correlation coefficient of −0.99, HSI was highly negatively correlated with GSI (*p* < 0.05).

### 2.2. Starvation during Ovarian Maturation Resulted in Chemical and Biochemical Changes in Hepatopancreas, Ovary and Serum

**Figure 2:**
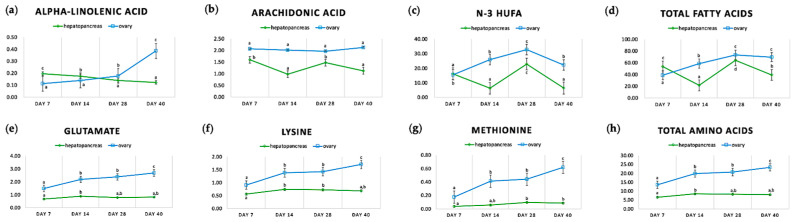
Dynamics of selected fatty acids and amino acids (% dry weight) in female *Scylla paramamosain* hepatopancreas and ovary under imposed starvation. (**a**) alpha-linolenic acid; (**b**) arachidonic acid; (**c**) n-3 HUFA; (**d**) total fatty acids; (**e**) glutamate; (**f**) lysine; (**g**) methionine; (**h**) total amino acids. HUFA, highly unsaturated fatty acids. Data are presented as mean ± SE (*n* = 3). Different letters on the same line indicate significant differences (*p* < 0.05).

We observed significant changes in the contents of fatty acids and amino acids in both the hepatopancreas and ovary throughout the starvation period (Figure 2). In general, the ovary contained much higher percentages of fatty acids and amino acids compared with the hepatopancreas. In the hepatopancreas, the total contents of fatty acids fluctuated and eventually decreased by 14.27% to 39.41% from day 7 to day 40. In the ovary, the total contents of fatty acids peaked at day 28 and dropped slightly to 69.63% at day 40. We only selected significant changes in fatty acids in both organs, as shown in Figure 2. The percentages of alpha-linolenic acid presented opposite trends with time, decreasing to 0.13% in the hepatopancreas in contrast to increasing to 0.39% in the ovary, suggesting that alpha-linolenic acid in the hepatopancreas might be translocated to the ovary for ovarian development against starvation. Arachidonic acid, however, only significantly fluctuated in the hepatopancreas, from 0.98% to 1.60%, but its fluctuation was not statistically significant in the ovary. The percentages of total n-3 highly unsaturated fatty acids (HUFA) consisting of DHA and EPA, reached a maximum at day 28 in both the hepatopancreas and ovary, at 22.90% and 32.80%, respectively, and decreased at day 40 back to their levels at day 14, at 6.46% and 22.34%, respectively. In terms of amino acids, the percentages of total amino acids, glutamate, lysine, and methionine all rose similarly until day 40 in both the ovary and hepatopancreas, which coincided with the changing HSI and GSI, possibly due to weight loss caused by starvation and therefore being less important to ovarian development.

During starvation, nutrients only come from endogenous sources, and therefore, serum biochemistry might reflect the utilization and transport of nutrients from the hepatopancreas and ovary. We found that serum lipids and serum glucose changed significantly with starvation time (Figure S1). Serum triglycerides peaked at day 14, reaching 0.16 mmol/L, and serum total cholesterol increased up to 0.31 mmol/L at day 40. Serum glucose was downregulated dramatically from 1.82 mmol/L at day 7 to 0.29 mmol/L at day 40. To further evaluate whether hepatopancreas function was affected by starvation, we assayed the hepatopancreas function transaminases in serum, glutamic pyruvic transaminase (ALT) and glutamic oxaloacetic transaminase (AST), which showed significant alterations in their enzyme activities with starvation time, with the activity of AST rising sharply, reaching 50 U/L at day 40 (Figure S1). The AST/ALT ratio can increase as hepatocellular injury occurs [19]. A significant elevation of the AST/ALT ratio from 0.56 on day 14 to 4.67 on day 28, which remained above 4 until day 40, indicated severe hepatocellular injury with an extended duration of imposed starvation from day 28 onwards.

### 2.3. The Landscape of the Scylla paramamosain Hepatopancreas and Ovary Transcriptomes

**Figure 3:**
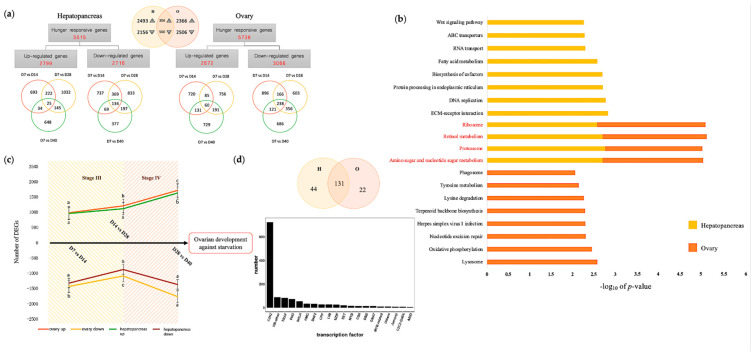
The landscape of *Scylla paramamosain* hepatopancreas and ovary transcriptomes. (**a**) Statistical comparisons of starvation-induced transcriptomic changes in the hepatopancreas (H) and ovary (O) tissues. Venn diagrams display the overlaps of differentially expressed genes in the hepatopancreas and ovary tissues of individual time points versus control, as well as that of the sum of all time points. (**b**) Gene set enrichment analysis of representative KEGG enrichment pathways enriched in hepatopancreas (yellow bar) and ovary (orange bar) under imposed starvation as selected by the lowest *p* values. (**c**) Time-specific gene expression trends. The numbers of upregulated (green line) plus downregulated genes (brown line) in the hepatopancreas and the numbers of upregulated (orange line) plus downregulated genes (yellow line) in the ovary are plotted against starvation time points. Data are presented as mean ± SE (*n* = 3). Different letters on the same line indicate significant differences (*p* < 0.05). (**d**) Comparison of starvation-responsive transcription factor (TF) genes in the hepatopancreas and ovary. The Venn diagram displays the number of identified TF genes in the hepatopancreas (H) and ovary (O). The bar chart presents the number of amino acid sequences corresponding to the TF family.

RNA-seq produced altogether 146.78 Gb clean data, with 2.05 × 10^7^ ± 1.68 × 10^6^ clean reads per sample. The Q30 was above 95.12% for all samples, indicating good quality. The average percentage of mapped reads to the *Scylla paramamosain* reference genome reached 82.43%, with a total of 9769 new genes detected, of which 6326 were functionally annotated. These results confirmed the quality of our data, which were appropriate for the downstream analyses. Using this dataset, 5515 differentially expressed genes (DEGs) from the hepatopancreas and 5738 DEGs from the ovary, corresponding to 28.05% and 26.98% of all genes annotated, respectively, were identified as displaying significant changes in gene abundance under imposed starvation to sustain ovarian development (Figure 3a).

It is noteworthy that the female mud crab hepatopancreas shared only 306 upregulated and 560 downregulated DEGs with the ovary, which account for merely 5.55% and 10.15% of hepatopancreas starvation-responsive genes, respectively, indicating obvious regional specificity. The regional specificity of gene expression was also clearly reflected by the principal component analysis in Figure S2 and the gene expression heatmap in Figure S3. Furthermore, gene set enrichment analysis (GSEA) was conducted to study the most significant KEGG enrichment pathways featuring tissue-specific signaling mechanisms induced by starvation, as shown in Figure 3b. Among the top 12 significantly enriched up-/downregulated pathways for each tissue, only 4 were common between hepatopancreas and ovary, which were “amino sugar and nucleotide sugar metabolism”, “proteasome”, “retinol metabolism”, and “ribosome”, while two-thirds of the most significant enriched pathways were tissue-exclusive. Hepatopancreas-exclusive differential gene functions were mainly associated with “ECM-receptor interaction”, “DNA replication”, and “protein processing in the endoplasmic reticulum” (yellow bars in Figure 3b), whereas ovary-exclusive functions were mainly associated with “lysosome”, “oxidative phosphorylation”, and “nucleotide excision repair” (orange bars in Figure 3b). As expected, other pathways, such as “fatty acid metabolism” for the hepatopancreas and “phagosome” for the ovary, were also significantly enriched.

Among the 974 DEGs induced by 14 days of starvation in the hepatopancreas, 247 (25.36%) and 59 (6.06%) DEGs were also upregulated in response to 28 and 40 days of starvation, respectively. Similarly, 38.43% and 15.51% of the DEGs negatively regulated by 7-day exposure to starvation were also downregulated in response to 28 and 40 days of starvation, respectively (Figure 3a). These results suggested that as the duration of starvation increased, only a small number of DEGs at the initial stage of starvation were also critical for adaptation to prolonged starvation in the mud crab hepatopancreas. Interestingly, a similar scenario was observed in the ovary. As shown in Figure 3a, among the 996 DEGs induced and 1421 DEGs suppressed by 7 days of starvation, only 14.56% and 19.18% of DEGs were also upregulated, while 28.43% and 25.26% were also downregulated in response to 28 and 40 days of starvation, respectively. Next, time-specific differentially expressed genes were analyzed by comparing adjacent time points (e.g., D7 vs. D14, D14 vs. d28, D28 vs. D40). We noticed a significant increase in upregulated DEGs in the D28 vs. D40 groups as the ovary matured under prolonged starvation, with 1637 DEGs in the hepatopancreas and 1722 DEGs in the ovary (Figure 3c). Numerically, these DEGs account for almost one-third of all DEGs, at 29.68% and 30.01%, respectively. This dramatic increase in upregulating genes suggested the profound mobilization of hepatopancreas and ovary genes to overcome long-term starvation and maintain ovarian maturation. GSEA analysis revealed that at the specific stage D28 vs. D40, the “FoxO signaling pathway” was significantly enriched in both hepatopancreas and ovary, with “FoxO signaling pathway”, “proteasome”, and “pentose and glucuronate interconversions” in hepatopancreas, as well as “lysosome”, “RNA transport”, and “lysine degradation” in the ovary as the most significant KEGG enrichment pathways.

Genes with high connectivity are generally located upstream of the regulatory network and are generally regulatory factors such as transcription factors (TFs). We, therefore, detected 197 TF-encoding genes differentially regulated by starvation stress across all time points in the hepatopancreas and ovary tissues, accounting for approximately 14.78% of the total of 1333 identified TF-encoding genes in female *Scylla paramamosain*. These starvation-responsive TF-encoding genes belonged to 39 diverse TF families, as shown in Figure 3d, representing different regulatory pathways. Among these TF families, we found that the C2H2 gene was the most abundant, with 725 genes, followed by HB-other (90), TRAF (84), and PHD (74). To further assess the differences in starvation-driven TF genes between the hepatopancreas and ovary tissues, we obtained 44 starvation-responsive TF genes that were up/downregulated in the hepatopancreas but not in the ovary and 22 TF genes that were up/downregulated in the ovary but not in the hepatopancreas (Figure 3d). Among these starvation-responsive TF genes, a large proportion (131 out of 197) was commonly regulated in both tissues, suggesting that, in general, hepatopancreas and ovary recruited similar transcription regulators in response to starvation. This indicated that the mud crab seems to rely mainly on C2H2 to maintain ovarian development against starvation in both the hepatopancreas and ovary.

Additionally, we identified six primary alternative splicing forms (alternative 5′ first exon, TSS; alternative 3′ last exon, TTS; alternative exon ends of 5′, 3′, or both, AE; skipped exon, SKIP; approximate alternative exon ends, XAE; and approximate SKIP, XSKIP) in mud crab hepatopancreas and ovary under imposed starvation. In both hepatopancreas and ovary, most were TSS and TTS events, at 44.47–45.66% and 37.08–40.71%, respectively, of all events. These alternative splicing events exhibited relatively stable profiles over time in both organs (Figure S4), indicating that tissues do not seem to rely on alternative splicing events to maintain ovarian maturation in starving conditions.

Taken together, these data collectively show that starvation dynamically affected gene expression in female *Scylla paramamosain* to sustain ovarian maturation in both temporal- and tissue-specific manners.

### 2.4. K-Means Analysis Revealed Time-Specific Gene Clusters

To decipher the interrelationships between starvation-responsive genes, we subjected the time-series expression of all DEGs to k-means clustering. This approach produced 10 clusters in the hepatopancreas and 10 clusters in the ovary, and some of these clusters might be linked to mud crab ovarian maturation under imposed starvation.

In the hepatopancreas, the largest cluster, cluster 5 (H-C5), containing 3404 DEGs, and the second largest cluster (H-C10), containing 2646 DEGs, remained downregulated until day 28 and kept a sharp increase until day 40 (Table S1). The KEGG pathways represented in this cluster mainly included “proteasome”, “RNA degradation”, and “autophagy—other”, emphasizing that autophagy also played a role in hepatopancreas over long-term starvation to sustain ovarian maturation. We also selected two typical clusters, H-C6 and H-C8, with a sharp induction of DEGs peaking at day 14 and day 28, respectively (Table S1), in which the KEGG pathway “biosynthesis of unsaturated fatty acids” was typically enriched earlier for day 14, whereas “lysosome” was typically enriched for day 28, showing time-specific expression patterns in mud crab hepatopancreas.

In the ovary, the largest cluster, cluster 8 (O-C8), containing 3927 DEGs, showed an early-phase repression pattern, with a rapid reduction from day 7 to day 14, which remained downregulated until day 40 (Table S1). The KEGG pathways represented in this cluster included various nutrition and metabolism pathways such as “sphingolipid metabolism”, “ubiquinone and another terpenoid-quinone biosynthesis”, “retinol metabolism”, “glycosphingolipid biosynthesis—globo and isoglobo series”, “linoleic acid metabolism”, “glycosphingolipid biosynthesis—lacto and neolacto series”, “starch and sucrose metabolism”, “amino sugar and nucleotide sugar metabolism”, “ascorbate and aldarate metabolism” as well as “other glycan degradation”. The second largest cluster (O-C1) exhibited an opposite trend, which kept downregulated until day 28 with a sudden rise of the expression level up to day 40 (Table S1). The significant KEGG pathways enriched in this cluster were “inositol phosphate metabolism” and “phosphatidylinositol signaling system”. Moreover, O-C6 (2703 DEGs) and O-C9 (2541 DEGs) showed a peak of expression level typically at day 14 and day 28, respectively (Table S1), comprising genes enriched in “ribosome”, “oxidative phosphorylation”, and “glutathione metabolism” for day 14, and in “lysine degradation” for day 28. These results suggested that in the early phase, various nutrition metabolisms in cluster 8 were suppressed in the ovary as starvation proceeded, and glutathione metabolism was turned on in the ovary to cope with oxidative stress caused by short-term starvation from day 7 to day 14. In the late phase until day 40, the phosphatidylinositol signaling system was induced in the ovary to activate ovarian development under prolonged starvation.

### 2.5. Starvation Alters the Expression of Genes Relevant to Epigenetic Modification

We analyzed the transcriptome in our RNA-seq datasets to identify three categories of epigenetic modifications, i.e., histone modification (744), RNA modification (12), and chromatin remodeling (4) over starvation. The most significant epigenetic modifications during long-term starvation from day 28 onwards involved RNA degradation, histone acetylation, and RNA splicing, coinciding with the sharp increase of DEGs during this period. Among these epigenetic regulators, 48 histone acetyltransferases were identified, which played a significant role in different organs. The KEGG pathways induced by histone acetyltransferases included “FoxO signaling pathway (ko04068)”, “Wnt signaling pathway (ko04310)”, “notch signaling pathway (ko04330)”, and “TGF-beta signaling pathway (ko04350)”. The expression levels of two significant histone acetyltransferases, CREB-binding protein-like isoform X1 and chromatin modification-related protein MEAF6-like (Figure S5), exhibited a slowly declining tendency until day 28 and a sharp increase up to day 40 in hepatopancreas. Whereas in the ovary, CREB-binding protein-like isoform X1 presented a rapid decrease until day 14 and a great increase up to day 40, yet chromatin modification-related protein MEAF6-like only fluctuated slightly over time. These data indicated that histone acetylation might play a key role in gene regulation to sustain ovarian maturation, especially over prolonged starvation.

### 2.6. Starvation-Responsive Genes Are Highly Linked to Tissue-Specific Traits

**Figure 4:**
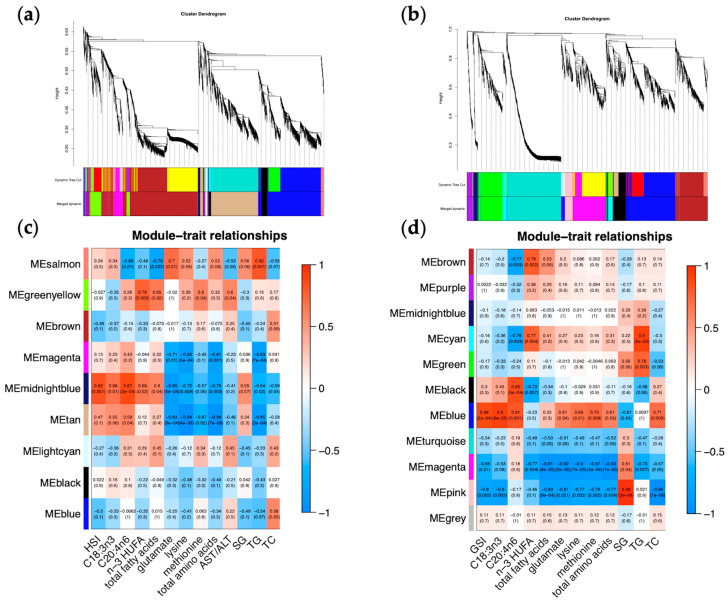
Weighted gene co-expression network analysis (WGCNA) of the hepatopancreas and ovary tissues in *Scylla paramamosain* under imposed starvation. (**a**,**b**) Gene dendrogram and module colors in hepatopancreas (**a**) and ovary (**b**) using the dynamic tree cut method. (**c**,**d**) Heatmaps show the correlation between identified modules and phenotype traits of the hepatopancreas (**c**) and ovary (**d**), where red represents positive correlation, blue represents negative correlation, upper numbers represent correlation coefficients, and numbers in parentheses represent the *p*-value.

In the hepatopancreas, we detected 9 modules that contained 3512 genes using the WGCNA algorithm (Figure 4a,c). A close examination of gene numbers within each module showed that the module size ranged from 33 genes in the light cyan module to 1144 genes in the brown module. Likewise, we identified 11 different co-expression modules in the ovary (Figure 4b,d), from 45 genes in the midnight blue module to 579 genes in the turquoise module, with the grey module representing genes with inadequate co-expression with other module genes. Subsequently, we tried to link the significantly different traits induced by starvation with each module and evaluated the relevance between them. As shown in Figure 4, 5 out of 9 hepatopancreas modules were significantly linked to the hepatopancreas traits, whereas 8 of the 11 ovary modules were significantly linked to the ovary traits, with |correlation| ≥ 0.75 and *p*-value ≤ 0.01. Specifically, the hepatopancreas-specific midnight blue and tan modules were highly correlated and significantly linked to four traits, including HSI, which was most significantly enriched in “steroid biosynthesis” and “phagosome”, respectively, whose gene expression levels remained downregulated as starvation proceeded. On the contrary, the blue and brown modules significantly enriched in “spliceosome” and “oxidative phosphorylation”, respectively, were weakly and negatively linked to HSI, and their gene expression patterns indicated that energy supply might be the cause of the decreased hepatopancreas mass over prolonged starvation. Moreover, the salmon module significantly enriched in “fatty acid biosynthesis” was upregulated only at day 14, featuring early-phase starvation, which was negatively linked to hepatopancreas total fatty acids and serum triglycerides and positively linked to serum triglycerides.

**Figure 5:**
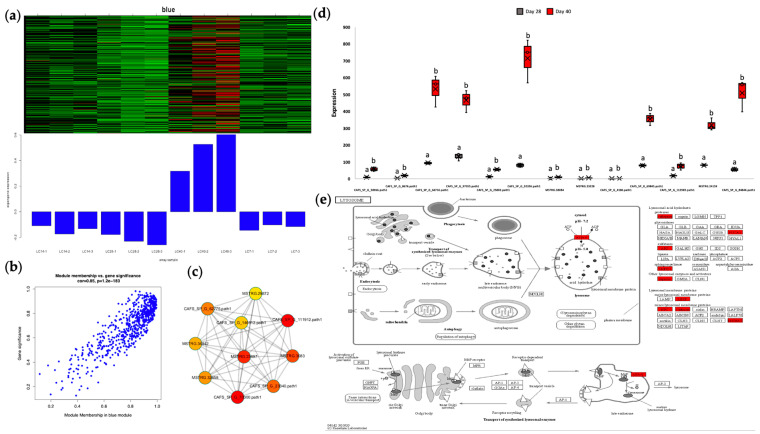
The ovary blue module. (**a**) Gene heatmap and epigengene expression bar chart within the module, with each row of the heatmap containing one gene and each column containing one sample. (**b**) Scatterplot of gene significance versus module membership. (**c**) The interaction network of the top 10 hub genes in the module. The color gradient from red to yellow corresponds to the rank from high to low. (**d**,**e**) The representative KEGG enrichment pathway of the module—Lysosome (ko04142). (**d**) Box-and-whisker of the difference in the abundance in FPKM of each gene enriched in the lysosome between day 28 and day 40, when the module was significantly upregulated. Different letters above the box of the same gene indicate significant upregulation (*p* < 0.05). (**e**) KEGG map of the lysosome. Genes with red boxes are significantly upregulated.

In the ovary, the blue module (Figure 5) with a total of 14 genes enriched and significantly unregulated in “lysosome (ko04142)” was highly expressed at day 40 (*p* < 0.05) featuring late-phase starvation, which was significantly positively linked to both GSI and ovary alpha-linolenic acid, as opposed to the pink module negatively linked to these two traits. The blue module was also positively linked to ovary arachidonic acid, while the pink module was also positively linked to serum glucose and negatively linked to the other six traits: ovary total fatty acids, ovary glutamate, ovary lysine, ovary methionine, ovary total amino acids, and serum total cholesterol. The magenta module highly correlated with the pink module and presented similar trait correlation patterns in terms of GSI, ovary fatty acids, and amino acids, and these two modules, which were upregulated during early-phase starvation from day 7 to day 14, were both enriched in the “autophagy-animal” pathway.

Therefore, we selected the ovary blue module (Figure 5) as the core gene module most relevant to mud crab ovarian development in terms of GSI increment under prolonged starvation, which was mainly enriched in the lysosome and autophagy-related genes. In order to further figure out the regulatory genes in the ovary blue module (Figure 5c), the interaction networks of the top 10 hub genes in this module were explored. Gene annotation results identified the CAFS_SP_G_111912.path1 in the ovary blue module as lysosomal Pro-X carboxypeptidase-like involved in protein digestion and absorption and the MSTRG.23697 (protein kinase), CAFS_SP_G_23340.path1 (protein FAN-like), and CAFS_SP_G_62775.path1 (protein crumbs-like isoform X1) to be involved in signal transduction mechanisms.

Collectively, these results showed that the gene expression network has captured core physiological processes at work within the hepatopancreas and ovary tissues of *Scylla paramamosain* under imposed starvation and the gene clusters that are likely responsible for them.

## 3. Discussion

### 3.1. Terminal Investment of Scylla paramamosain into Ovarian Maturation

Crustaceans have a more persistent tolerance to starvation than fish. A previous study demonstrated that the Chinese mitten crab, *Eriocheir sinensis*, survived a 42-day starvation experiment [20]. In our experiment, female *Scylla paramamosain* successfully survived 40 days of starvation.

Starvation is a common challenge in both natural and cultural environments. In nature, overwintering female *Scylla paramamosain* usually suffers from prolonged starvation while sustaining ovarian maturation. Under the intensive pond arrangement and paddy field integrated culture mode, the mud crabs often suffer body starvation due to nutritional limitations (life cycle restrictions, food availability, and food quality), high stocking density, intraspecific fighting, and such harsh environments [6,18], resulting in reduced reproduction. Food availability/limitation is an important aspect of environmental heterogeneity [21], when the allocation of limited resources lies at the heart of life history trade-offs [10]. Evolutionary theory predicts that as an individual is exposed to a threat to future reproduction upon its perception of increased likelihood of mortality, energy allocation should be triggered in favor of immediate reproduction, and such reproductive effort has been termed the terminal investment hypothesis/fecundity compensation [22]. We observed increased ovarian maturation under imposed starvation in female *Scylla paramamosain,* indicating plasticity of energy allocation toward reproductive investment. Starvation indeed serves as a cue to induce an individual’s propensity for terminal investment. For example, reproductive adaptation in adult morphs of *Sitobion avenae* under starvation stress contained more mature embryos than those starved for shorter periods, with the former tending to invest in the development of larger embryos at the expense of reducing lifespan [23]. Food-deprived yellow mealworm beetle *Tenebrio molitor* elicits investment in their sexual attractiveness at the expense of their survival [24]. In humped-winged grigs, females were more quick to remate when held on a low-quality diet [25]. However, on the other side, there have also been studies demonstrating that food limitations lead to decreased reproductive effort due to a lack of essential nutrients. For example, cockroaches (*Nauphoeta cinerea*) have a significantly shorter reproductive lifespan when reared on a low-quality diet [26].

The terminal investment of *Scylla paramamosain* in our study showed that overall, GSI and HSI were significantly inversely related, suggesting the resorption of hepatopancreas to develop ovary. WGCNA analysis also indicated that “oxidative phosphorylation” might be the cause of the decreased hepatopancreas mass over prolonged starvation. As a consequence of starvation, a decline in the hepatosomatic index has also been observed in the crab species *Callinectes danae*, *C. ornatus*, *Lithodes santolla* [8], *Eriocheir sinensis* [27], and shrimp species [28], which use part of their reserves stored in the hepatopancreas to develop the reproductive system in the starvation treatment compared with ad libitum, especially in female crabs. Thus, there is a trade-off existing between somatic cells and sex cells to resist starvation. During sexual reproduction, resources are switched to reproduction instead of somatic cell proliferation/maintenance. Increased future reproduction at the cost of decreased somatic maintenance has also been observed in *Caenorhabditis elegans* and *Drosophila melanogaster* [29].

In aquaculture, terminal investment in ovarian maturation under imposed starvation shows application potential with its advantages of ovarian development maintenance in a shortage of food. Furthermore, according to the life history theory, such terminal investment triggered by starvation is vital not only for successful sexual reproduction but also for the long-term survival of an organism or even a species [30]. On the other side, we observed a slower *Scylla paramamosain* ovarian development process that took as long as 28 days to reach ovary stage IV and failed to develop into stage V even after 40 days. Therefore, although food limitations did not pause ovarian development, they did show disadvantages in the slower development process and poorer ovarian quality. Histology in Figure 1b also showed that prolonged starvation may elicit negative autophagic effects with clear ovary shrinkage.

### 3.2. Phase Specificity of Ovarian Development under Imposed Starvation

According to the ovarian development stage, we divided the ovarian development under starvation into a two-step process, with starvation days 7–14 as the early phase starvation and starvation days 28–40 as the late phase starvation. From day 14 to day 28, the oocyte matured from stage III to IV. The increment in ovary mass was not significant, but the increment in oocyte diameter was significant, indicating all energy was allocated to oocyte transformation.

Starvation studies on the usage of carbohydrates, lipids, and proteins may be useful predictors to determine energetic and metabolic requirements [18]. In our study, fatty acids were primarily transferred as a source of ovarian development from the hepatopancreas, which has always been a vital organ involved in a variety of metabolic activities, including nutrient supply [31]. During early phase starvation with the development of stage III ovary, fatty acids such as alpha-linolenic acid and n-3 HUFA might transfer from the hepatopancreas to the ovary, which might be important nutrients to promote ovarian development. During late-phase starvation with the development of stage IV ovary, total fatty acids and n-3 HUFA in both hepatopancreas and ovary declined with time, except alpha-linolenic acid, which might still transfer from hepatopancreas to ovary. Therefore, the lack of essential n-3 HUFA to support ovarian development from the hepatopancreas in the late starvation phase might account for the prolonged development of ovarian stage IV and probable failure into stage V. This was also true in *Eriocheir sinensis* during the duration of starvation; the hepatopancreas index significantly decreased with lipid metabolism dominating, thus activating the fatty acid metabolism pathway in vivo [20]. Moreover, we identified that during early phase starvation, serum triglyceride was also increasing with time, indicative of lipid mobilization from the hepatopancreas to the ovary. In k-means and WGCNA analysis, the hepatopancreas was significantly enriched in “biosynthesis of unsaturated fatty acids” and “fatty acid biosynthesis” in the early phase, possibly to supply essential fatty acids for the ovary, which was no longer significant in the late phase starvation.

### 3.3. Autophagy at the Core of Regulatory Pathways

**Figure 6:**
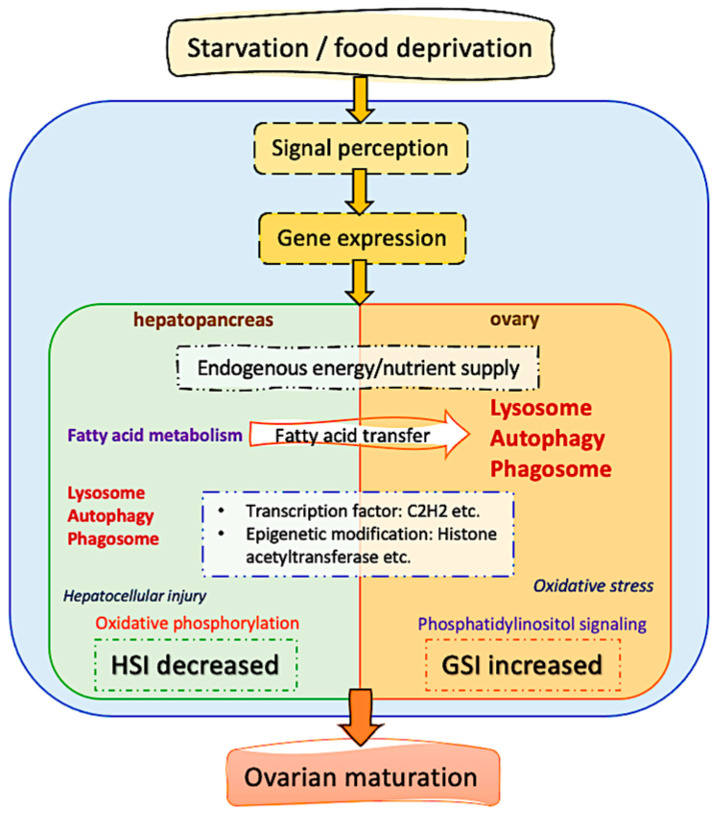
A schematic representation of major processes involved in differential anti-starvation regulations in the hepatopancreas and ovary tissues in female *Scylla paramamosain*.

A schematic representation of major processes involved in differential anti-starvation regulations in the hepatopancreas and ovary tissues in female *Scylla paramamosain* was illustrated in Figure 6. Enrichment analysis from GSEA, k-means, and WGCNA all revealed that autophagy-related pathways such as “lysosome”, “autophagy-animal”, and “phagosome” were key pathways in both organs of female *Scylla paramamosain* at various starvation time points responsible for ovarian development under starvation, as correlated with GSI increment. In the hepatopancreas, cells might be consumed through apoptosis to maintain energy metabolism. This is also the case in *Eriocheir sinensis*, which selects to consume hepatopancreatic cells as triggered by the death receptor pathway, with autophagy increased in the hepatopancreas [20]. In the ovary, starvation-induced autophagy was induced to promote ovarian maturation, as we confirmed. Histology in Figure 1b showed that starvation stimulated ovarian maturation, possibly through an increased autophagic process in vitellogenic oocytes. Comparing the species studied so far, starvation could also induce oocyte maturation associated with the upregulation of autophagy markers in the ovary tissue of the female prawns, which could drive vitellin utilization [32]. Autophagy participates in Drosophila’s reproduction by primarily mediating appropriate cell death to provide resources for oogenesis [33]. In *Daphnia pulex*, females resorb meiotic oocytes to resist starvation [34]. In the endoparasitic wasp, *Pteromalus puparum*, vitellin in the oocytes was decomposed and transported into the hemolymph [35].

Autophagy itself not only maintains the normal physiological function of cells in a basal state but also serves as an adaptive process induced by stresses such as starvation, infection, and hypoxia [36,37]. Autophagy was also found to be an important regulator for resource allocation during reproduction, including spermatogenesis, oogenesis, fertilization, embryogenesis, and others [38]. As an evolutionarily conserved process, autophagy provides a way to ‘eat ourselves,’ which delivers cytoplasmic material to the lysosome for degradation to provide recycled materials for further cellular renovation or even the survival of cells under stress conditions [39]. The signaling pathway regulating autophagy is very complex. In addition to the 40 autophagy-related genes involved in the formation of autophagosomes, there are TFs that participate in the regulation of autophagy [40,41]. To cope with starvation, the upregulated TF C2H2 gene family was overrepresented in our study since zinc-finger protein can participate in various biological processes such as apoptosis, autophagy, autophagic degradation, and the response to stress [42,43]. Moreover, the FoxO signaling pathway was also significantly enriched in our study. It is noteworthy that the phosphoinositide 3-kinase/protein kinase B/Forkhead box O (PI3K/PKB/FOXO) signaling pathway has been previously reported to be an important upstream regulatory mechanism for autophagy [44]. Furthermore, epigenetic modifications have various protective roles during starvation [45,46]. Under nutritional stress, several histone marks have been reported that may act as switches upon starvation for stress-response pathways [47]. We observed upregulated histone acetylation under prolonged starvation in both organs, which has also been reported in the regulation of autophagy [48]. Histone acetyltransferase transfers the acetyl group of acetyl CoA to the lysine residue of histones, neutralizing the positive charge of histones, weakening the interaction between DNA and histones, and making it easier for DNA to bind to TFs [49]. The cyclic AMP (cAMP) response element binding (CREB) protein gene, CREB-binding protein-like isoform X1, was the most dominant in our study. The histone acetyltransferase CREBBP/CBP also promotes autophagosome–lysosome fusion in the late stage of autophagy in previous studies [50]. In *Caenorhabditis elegans*, the TF CREB is activated by starvation [51]. Starvation resistance in *Drosophila melanogaster* was also regulated through CREB-regulated transcription coactivators [52].

## 4. Materials and Methods

### 4.1. Ethics Statement

All animal experiments in this study were conducted in accordance with the relevant national and international guidelines. Our project was approved by the East China Sea Fisheries Research Institute. In China, catching wild mud crabs in seawater does not require specific permits. Our study did not involve endangered or protected species.

### 4.2. Animal Materials and Starvation Experiment

As sourced from Ninghai Town, Zhejiang Province, China, in June 2023, only female, uninjured egg-bearing mud crabs (*Scylla paramamosain*) were selected, held in aquariums, and fed ad libitum on razor clams until hatching. Altogether, 12 female mud crabs were selected immediately after hatching, weighing 400 ± 10.94 g, with Fulton’s condition factor at 0.62 ± 0.05 and the female maturity index at 1.04 ± 0.02. Each crab was maintained in a separate tank to avoid cannibalism and simultaneously randomized into four sets consisting of three replicates, each to meet the validity of the statistical model and the robustness of the experimental results. Feeding was stopped immediately after hatching (day 0), and the four sets of crabs were correspondingly sampled on days 7, 14, 28, and 40. The first 7 days of starvation were for a gut cleanse to deplete old food and chemical reserves accumulated in the crab digestive system. Therefore, we took “Day 7” as the starting point of the starvation experiment after the depletion of food reserves and also as the control group in this longitudinal study. The temperature was set at 21–23 °C and the salinity at 24–26‰. At each sampling time, the particular set of crabs was dissected alive on ice, and the ovary, hepatopancreas, and hemolymph samples of each individual crab were collected for multiple analyses, as specified below.

### 4.3. Hemolymph Collection and Serum Biochemistry

A total of 12 hemolymph samples were collected in 1.5 mL Eppendorf at each sampling time with three biological replicates, mixed with anticoagulant 10% potassium oxalate, and centrifuged at 4 °C at 3000 rpm for 5 min. The supernatant was collected as a serum for a biochemistry assay measured by a HITACHI 3500 automatic analyzer (Chiyoda City, Japan). The biochemical parameters include globulin (g/L), alanine aminotransferase (U/L), aspartate aminotransferase (U/L), alkaline phosphatase (U/L), glutamine transpeptidase (U/L), total protein (g/L), albumin (g/L), glucose (mmol/L), triglycerides (mmol/L), and total cholesterol (mmol/L).

### 4.4. Tissue Collection and Histology

Altogether, 12 ovary tissues from each crab were isolated for HE staining. After rinsing with 1 × PBS, the tissues were blotted dry with filter paper and weighed. A pea-sized piece of each tissue was transferred to Bonn fixation for about 24 h and then washed with 70% alcohol three times before putting it into 70% alcohol for −20 °C preservation. Paraffin slices were stained and dehydrated as previously described [53]. The histology results were then interpreted. A previous study confirmed all mud crabs immediately after hatching were at the same starting point of the early vitellogenesis stage (stage III, ovarian color light-yellow) [53]. The ovarian stages in our study were distinguished as the early vitellogenesis stage (stage III, light-yellow) and the late vitellogenesis stage (stage IV, orange) [54,55], as confirmed by both morphology and histology.

### 4.5. Chemical Analyses

An adequate amount of the tissues from the ovary (12 samples) and hepatopancreas (12 samples) of each crab were washed with freshwater before fatty acid and amino acid analysis.

#### 4.5.1. GC–MS Analysis of Fatty Acids

The 24 samples were hydrolyzed and extracted as previously described [54]. A total of 100 μL upper clear liquid was diluted by n-hexane to 1 mL and filtered by a 0.45 μ membrane. The data collection instrument system was Thermo Trace 1310 ISQ (Thermo Fisher Scientific, Waltham, MA, USA), with HP-88 Agilent 100 m × 0.25 mm × 0.20 μM as chromatographic column, injection port temperature at 290 °C, and He as carrier gas. The flow rate was set at 1.0 mL/min. The heating program was initially maintained at 100 °C for 13 min, and the temperature was raised at a rate of 10 °C/min to 180 °C for 6 min, raised at a rate of 1.5 °C/min to 192 °C for 6 min, and then raised at a rate of 3.5 °C/min to 240 °C for 4 min. The integral peak area ratio of all detected samples of fatty acids was taken into the standard curve linear equation for calculation and further incorporated into the calculation formula to obtain the absolute data on fatty acid content. Fatty acid concentrations were expressed on a % dry basis.

#### 4.5.2. HPLC Analysis of Amino Acids

A total of 0.2 g of each sample was hydrolyzed and made into derivatives, as previously described [53]. A total of 0.45 mL of mobile phase A, consisting of 0.1 mol/L anhydrous sodium acetate and acetonitrile, was added, mixed well, and passed through the membrane. The operating analysis conditions were C18 chromatographic column, 1 mL/min flow rate, 40 °C column temperature, 10 μL injection volume, and a 254 nm UV detector. The amino acid contents were calculated using the sample volume after hydrolysis, and the concentration of each amino acid was calculated on the standard curve. Amino acid concentrations were converted to % dry basis as well.

### 4.6. Statistical Analysis

Statistical analyses of the above-mentioned biochemical and physiological traits in hepatopancreas, ovary, and serum were performed with SPSS 26.0 software, with three biological replicates for each time group. The Q-Q plot and Levene’s test showed that all data were distributed normally and homoscedastically. The significant differences among treatments at *p* < 0.05 were statistically evaluated using the LSD-*t* test.

### 4.7. RNA Isolation, Library Preparation, and RNA-Seq

To characterize the starvation response at the molecular level, all 24 samples were used for RNA-seq as previously described [53]. Quality inspection and quantification of products using Qubit fluorescence quantification/enzyme labeling instruments ensured that the RNA samples were subjected to quality control in three aspects: total quantity, purity, and completeness. Next, the double-stranded target region library was denatured, cyclized, and digested to obtain single-stranded circular DNA, which was amplified using rolling circle amplification technology to obtain DNA nanoballs. After the library construction was completed, Qubit (Thermo Fisher Scientific, Waltham, MA, USA) was used for quantitative quality control before machine sequencing. The sequencing instrument was the HUADA DNBSEQ-T7 gene sequencer (Mgi Tech Co., Shenzhen, China). DNA nanoballs were loaded onto a patterned array chip, and combinatorial probe-anchor synthesis was used for sequencing to finally obtain the original sequencing data through multiple cycles.

### 4.8. Sequence Alignment and Functional Annotation

Sequence alignment was performed between the clean reads of the 24 samples and the designated reference genome of *Scylla paramamosain,* as reported previously [56]. Only reads with a perfect match or one mismatch were further analyzed and annotated based on the reference genome using Hisat2 tools. Gene function was annotated based on the following databases: Nr (NCBI non-redundant protein sequences), Nt (NCBI non-redundant nucleotide sequences), Pfam (protein family), KOG/COG (clusters of orthologous groups of proteins), Swiss-Prot (a manually annotated and reviewed protein sequence database), KO (KEGG ortholog database), and GO (gene ontology). For the genes with no name in the genome GFF file, we used gene ID only.

### 4.9. Analysis of Gene Expression Patterns

A total of 24 samples were tested in our study, including four hepatopancreas groups and four ovary groups with three biological replicates per group. Quantification of gene expression levels was estimated by fragments per kilobase of transcript per million fragments mapped (FPKM). Subsequently, differentially expressed genes were identified based on their expression levels in different samples using the DESeq2 R package (1.26.0) based on the negative binomial distribution. The resulting *p*-values were adjusted using the Benjamini–Hochberg approach for controlling the false discovery rate. Genes with FDR < 0.05 and |log2(foldchange)| ≥ 1 were assigned as differentially expressed. GO enrichment analysis of the DEGs was implemented by the GOseq R packages based on the Wallenius non-central hyper-geometric distribution [57], which can adjust for gene length bias in DEGs. KEGG [58] is a database resource for understanding high-level functions and utilities of the biological system (http://www.genome.jp/kegg/ (accessed on 10 October 2023)). We used KOBAS 3.0 [59] software to test the statistical enrichment of differential expression genes in KEGG pathways, with a corrected *p*-value < 0.05. The GSEA analysis used the KEGG pathway and the gene sets of the BP, CC, and MF branches of GO as the sets of genes of interest and used the log_2_FC of each differential group as the score of the background gene set to analyze the enrichment of the set of genes of interest. We used the transcription factor database AnimalTFDB to classify and annotate whole genome TFs and transcription cofactors in our data. K-means clustering was analyzed using Mfuzzy Packets to obtain the temporal signature of gene expression and to divide different clusters [60].

### 4.10. Identification of Gene Modules Related to Ovarian Development under Imposed Starvation

Weighted gene co-expression network analysis (WGCNA, [61]) of the normalized expression (FPKM) of all the genes expressed was used to identify clusters of highly correlated transcriptomic modules within each tissue studied and then relate their expression patterns to hepatopancreas and ovary physiological variations, with the following parameters: the power was 1 (hepatopancreas) and 6 (ovary), the weighted network was unsigned, the minimum module size was 30, and the minimum height for merging modules was 0.25. Modules were combined with the protein–protein interaction (PPI) network from a search tool for retrieval of interacting genes/proteins (STRING) database (http://string-db.org/ (accessed on 20 October 2023)) to obtain the predicted PPI of these DEGs. Then, the PPI of these DEGs was visualized in Cytoscape [62]. The top ten intramodular hub genes were identified using Cytohubba with the MCC method [63].

## 5. Conclusions

Our study is the first to report the effects of starvation on endogenous ovary maturation in the mud crab *Scylla paramamosain*. In our experiment, female *Scylla paramamosain* successfully survived 40 days of starvation with ovarian maturation efforts at the expense of hepatopancreas mass. Under imposed starvation, ovarian tissue structure, fatty acid compositions, and serum biochemistry underwent adaptive changes in a two-step process, with hepatopancreas fatty acid supply to the ovary from day 7 to day 14 and autophagy activation in both organs from day 28 to day 40. Based on the above results, we propose an ovarian development strategy for food-limited female crustaceans involving the possession of (1) higher amounts of fatty acid stored in the hepatopancreas for nutrient supply and (2) activation of autophagy-related pathways for terminal investment in reproduction. Future studies with a better understanding of the mechanistic relationship between autophagy and ovarian maturation will open the door to new strategies to improve the nutritional and economic value of female crustaceans under starvation stress.

## Data Availability

Data is unavailable due to privacy.

## Supplementary Materials

The supporting information can be downloaded at: https://www.mdpi.com/article/10.3390/ijms25020700/s1.
