# Supplementary material for: Temporal and Spatial Signatures of Scylla paramamosain Transcriptome Reveal Mechanistic Insights into Endogenous Ovarian Maturation under Risk of Starvation
Source: Int J Mol Sci. 2024 Jan 5;25(2):700. doi: 10.3390/ijms25020700 (PMC10815400; doi:10.3390/ijms25020700)
Supplement: Supplementary file 1 [file ijms-25-00700-s001.zip › ijms-2753569-supplementary.pdf]

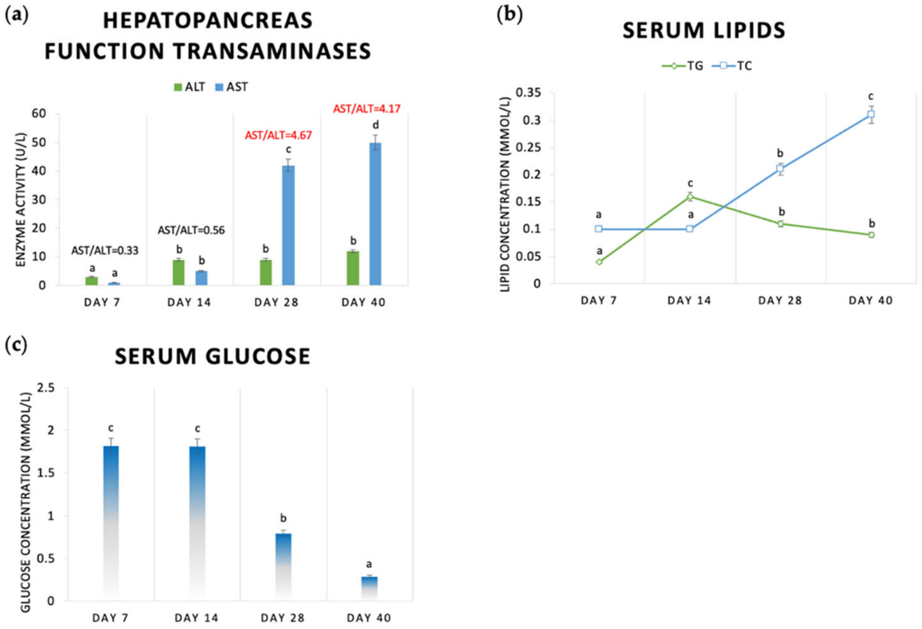

Figure S1. Dynamics of selected serum biomarkers in female *Scylla paramamosain* under imposed starvation. (**a**) hepatopancreas function transaminases; (**b**) serum lipids; (**c**) serum glucose. ALT, glutamic-pyruvic transaminase; AST, glutamic oxaloacetic transaminase; TG, serum triglycerides; TC, serum total cholesterol. Data are presented as mean ± SE (n=3). Different letters of the bar/line with the same color indicate significant difference (*P*< 0.05).

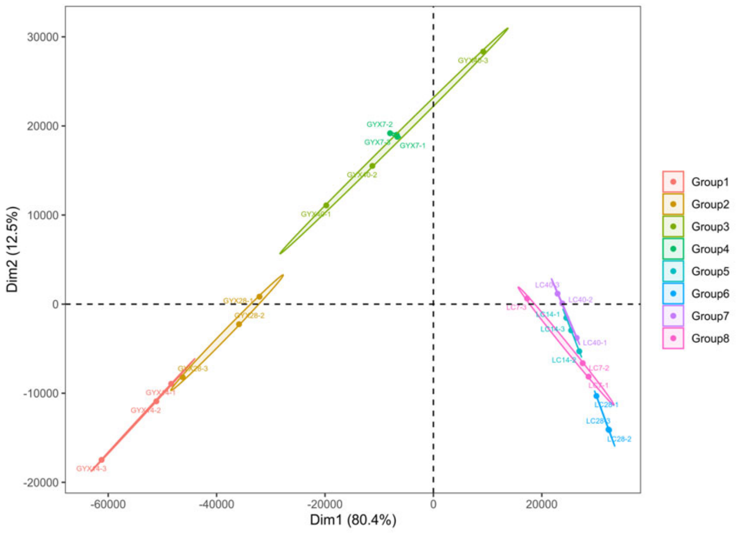

Figure S2. Principal component analysis of gene expression for all samples.

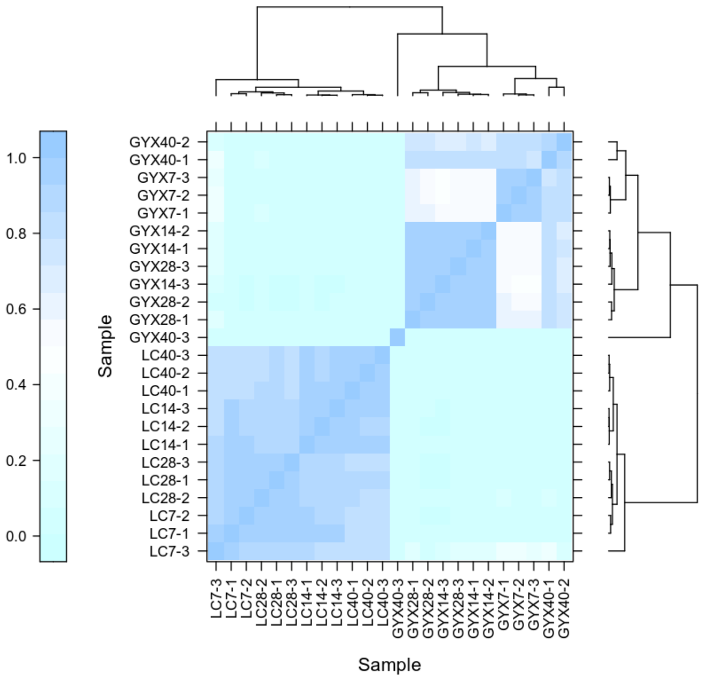

Figure S3. Heatmap of the mosaic gene expression patterns between each two samples with the clustering results of sample correlation. Use Pearson's Correlation Coefficient r as an evaluation indicator for biological repeated correlations. The closer the absolute value of R is to 1, the stronger the correlation between two samples.

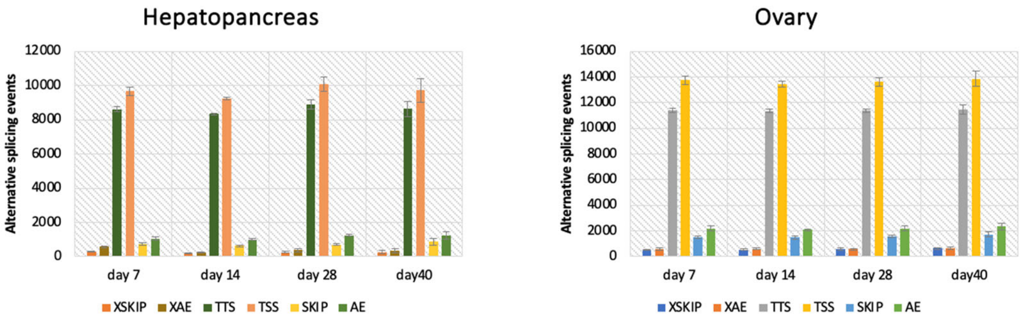

(**a**) (**b**)

Figure S4. Specific patterns of alternative splicing events in mud crab (**a**) hepatopancreas and (**b**) ovary under imposed starvation. Colors indicate different alternative splicing events. Data are presented as mean ± SE (n=3).

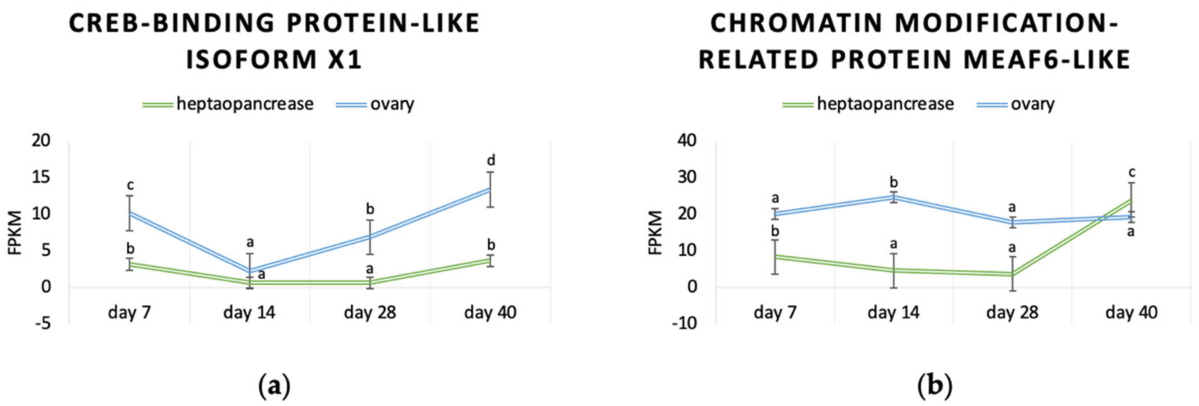

Figure S5. Features of epigenetic modifications. Temporal expression trends of (**a**) CREB-binding protein-like isoform X1 and (**b**) chromatin modification-related protein MEAF6-like in *Scylla paramamosain* hepatopancreas (green line) and ovary (blue line) under imposed starvation. Data are presented as mean ± SE (n=3). Different letters of the same line indicate significant difference ($P< 0.05$).

Table S1. Selective clusters with diverse patterns in hepatopancreas and ovary of female *Scylla paramamosain* under imposed starvation

Cluster name	Number of DEGs	KEGG enrichment (Corrected *P*-value <0.05)	Organ	Expression trends (color from orange to red indicates gene cluster membership)
**H-C5**	3404	D-Glutamine and D-glutamate metabolism;<br>mTOR signaling pathway;<br>RNA transport;<br>Arginine biosynthesis;<br>Longevity regulating pathway - multiple species	Hepatopancreas	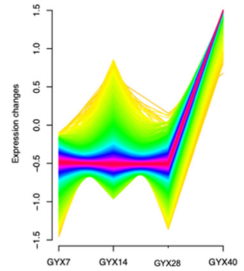
**H-C10**	2646	Proteasome;<br>RNA degradation;<br>Autophagy – other;<br>Basal transcription factors;<br>RNA polymerase;<br>Spliceosome	Hepatopancreas	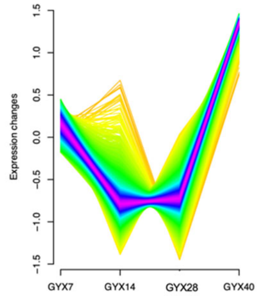
**H-C8**	2365	Ribosome;<br>Other glycan degradation;<br>Sphingolipid metabolism;<br>Lysosome;<br>Glycosphingolipid biosynthesis - globo and isoglobo series	Hepatopancreas	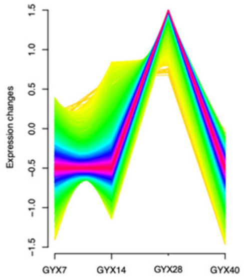

**H-C6**	1817	Ribosome;<br>Ubiquinone and other terpenoid-quinone biosynthesis;<br>RNA transport;<br>Phenylalanine metabolism;<br>Biosynthesis of unsaturated fatty acids;<br>Tyrosine metabolism	Hepatopancreas	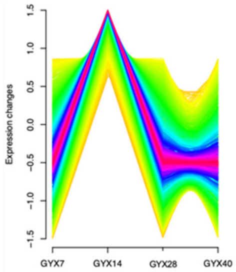
**O-C8**	3927	Lysosome;<br>Sphingolipid metabolism;<br>Ubiquinone and other terpenoid-quinone biosynthesis;<br>Retinol metabolism;<br>Glycosphingolipid biosynthesis - globo and isoglobo series;<br>Linoleic acid metabolism;<br>Glycosphingolipid biosynthesis - lacto and neolacto series;<br>Starch and sucrose metabolism;<br>Amino sugar and nucleotide sugar metabolism;<br>Ascorbate and aldarate metabolism;<br>Other glycan degradation	Ovary	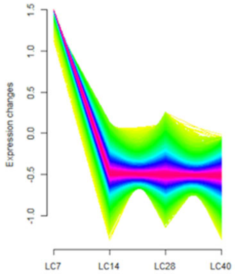
**O-C1**	2765	Inositol phosphate metabolism;<br>Phenylalanine metabolism;<br>Phosphatidylinositol signaling system	Ovary	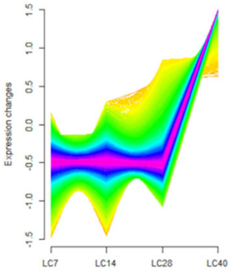
**O-C6**	2703	Ribosome;<br>Oxidative phosphorylation;<br>Glutathione metabolism	Ovary	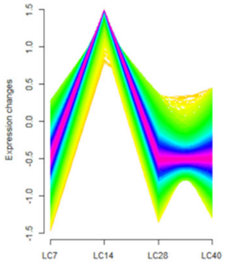
**O-C9**	2541	Lysine degradation	Ovary	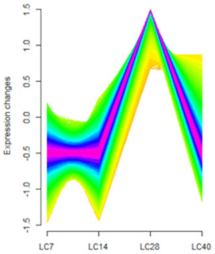
